# *Hummingbird*: monitoring and analyzing flash X-ray imaging experiments in real time^1^

**DOI:** 10.1107/S1600576716005926

**Published:** 2016-04-18

**Authors:** Benedikt J. Daurer, Max F. Hantke, Carl Nettelblad, Filipe R. N. C. Maia

**Affiliations:** a Laboratory of Molecular Biophysics, Department of Cell and Molecular Biology, Uppsala University, Sweden; b Division of Scientific Computing, Department of Information Technology, Science for Life Laboratory, Uppsala University, Sweden

**Keywords:** X-ray free-electron lasers, XFELs, flash X-ray imaging, real-time data analysis, computer programs

## Abstract

Advances in X-ray detectors and increases in the brightness of X-ray sources combined with more efficient sample delivery techniques have brought about tremendous increases in the speed of data collection in diffraction experiments. Using X-ray free-electron lasers such as the Linac Coherent Light Source (LCLS), more than 100 diffraction patterns can be collected in a second. These high data rates are invaluable for flash X-ray imaging (FXI), where aerosolized samples are exposed to the X-ray beam and the resulting diffraction patterns are used to reconstruct a three-dimensional image of the sample. Such experiments require immediate feedback on the quality of the data collected to adjust or validate experimental parameters, such as aerosol injector settings, beamline geometry or sample composition. The scarcity of available beamtime at the laser facilities makes any delay extremely costly. This paper presents *Hummingbird*, an open-source scalable Python-based software tool for real-time analysis of diffraction data with the purpose of giving users immediate feedback during their experiments. *Hummingbird* provides a fast, flexible and easy-to-use framework. It has already proven to be of great value in numerous FXI experiments at the LCLS.

## Abstract 2

*Hummingbird* is an open-source scalable Python-based software tool for real-time analysis of diffraction data with the purpose of giving users immediate feedback during their experiments.

## 1. Introduction   

More than a decade ago, it was first suggested that the very short and intense pulses of X-ray free-electron lasers (XFELs) have the potential to determine the structure of noncrystalline bioparticles from a large collection of single-shot diffraction patterns (Neutze *et al.*, 2000 ▸). This flash X-ray imaging (FXI) concept has been used to reconstruct two-dimensional projections of mimivirus (Seibert *et al.*, 2011 ▸), whole cells (van der Schot *et al.*, 2015 ▸), cell organelles (Hantke *et al.*, 2014 ▸) and soot particles (Loh *et al.*, 2012 ▸). More recent advances include the characterization of the three-dimensional structure of silver particles (Barke *et al.*, 2015 ▸) and even a full three-dimensional reconstruction of mimivirus (Ekeberg *et al.*, 2015 ▸). In a typical FXI experiment, a stream of biological particles is injected into the focus of a pulsed X-ray source. Far-field diffraction data are collected downstream of the interaction region. A more detailed description of the experimental setup, using an aerosol particle injector, has been given for example by Hantke *et al.* (2014 ▸).

Currently, a considerable amount of time during FXI experiments is spent on alignment and optimization of the X-ray source, background reduction and finding the intersection between the particle stream and the X-ray beam. These tasks get increasingly harder as we move towards harder X-rays (>2 keV) in a smaller focus (<200 nm), with ever smaller bioparticles (<100 nm). Yet such changes are necessary steps towards the goal of imaging single molecules with atomic resolution using a free-electron laser. The availability of immediate feedback on the quality of the data collected helps reduce the amount of time required for the above steps. For this purpose we developed *Hummingbird*, an open-source, modular and scalable Python-based software tool designed for monitoring and analyzing FXI experiments in real time. Similar projects like *Cheetah* (a comprehensive data reduction and analysis tool for diffraction data written in C/C++; Barty *et al.*, 2014 ▸), *CASS* (a data analysis and visualization tool developed for XFEL experiments; Foucar *et al.*, 2012 ▸), *cctbx.xfel* [a Python-based data processing tool for serial femtosecond crystallography (SFX); Sauter *et al.*, 2013 ▸] and *OnDA* (a real-time monitoring tool mainly developed for the needs of SFX; Mariani, 2016 ▸) show the need for robust and fast data analysis tools able to cope with the large stream of data produced with XFELs.

*Hummingbird* abstracts away the technical details required to access data at different light sources in a scalable manner, presenting the user with a consistent interface for processing individual shots. It is lightweight and can scale to processing rates above 100 Hz by distributing the load among multiple workers on multiple computation nodes. Its Python-based configuration files make it simple to use, while allowing great flexibility. We demonstrate the versatility and speed of *Hummingbird* using an example that reflects typical data taken at FXI experiments. Although *Hummingbird* has been developed and tested mainly for experiments performed at the Linac Coherent Light Source (LCLS), its modular architecture allows it to be easily adapted to any other light source.

## 2. Architecture   

**Figure 1:**
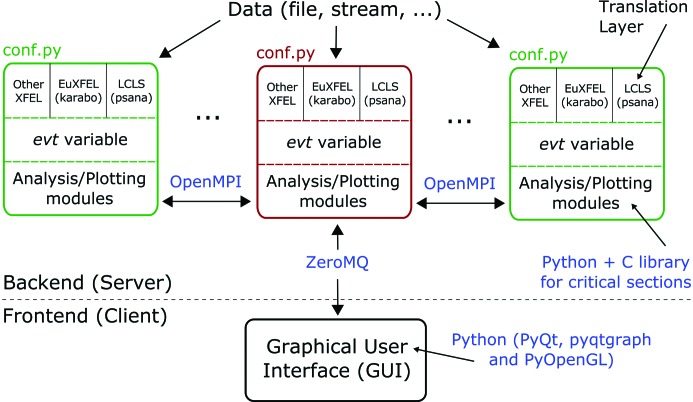
The client–server architecture of *Hummingbird*. Depending on the configuration (conf.py), the backend workers (master in red, slaves in green) read in data, translate native events based on the facility, perform user-specific data analysis and send plots off to the frontend.

*Hummingbird*’s architecture (see Fig. 1 ▸) is based on a simple client–server model written mainly in Python. The server can be split over multiple processes, communicating across MPI (Forum, 1994 ▸) using OpenMPI (Gabriel *et al.*, 2004 ▸), to maximize performance. Each process reads independently from a common data source, translating raw data into *Hummingbird*’s common event structure. For every event, user-specific data analysis is performed. The server communicates the results to the client using ZeroMQ’s publish/subscribe pattern (Hintjens, 2010 ▸). The client subscribes to available data sources and output is visualized in a graphical user interface (GUI).

**Figure 2:**
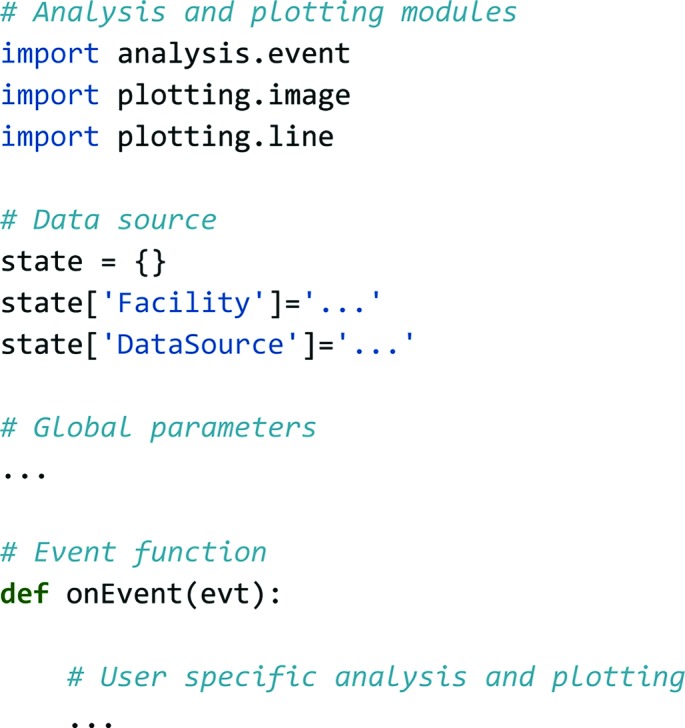
The backbone structure of a *Hummingbird* configuration file. In the header, analysis/plotting modules are imported, accompanied by a definition of the data source. Inside the event function, user-specific data analysis and plotting is defined. Global parameters can be defined outside the event function.

Customization of the backend is possible through a Python-based configuration file, as shown in Fig. 2 ▸, which specifies the data source and defines the analysis and plotting modules to be used. Upon changes, the configuration can easily be reloaded by sending a signal to the server, either using the terminal or remotely using the ‘reload’ button in the GUI. This makes the common procedure of tuning parameters, such as hit-finding thresholds, convenient and fast.

This section describes the individual parts of *Hummingbird*’s architecture, namely the configuration file, translation layer, event variable, analysis/plotting modules and GUI. A complete example based on experimental data followed by a simple benchmark to test *Hummingbird*’s speed and scalability is given in §3.

### 2.1. Configuration file   

A typical *Hummingbird* configuration file (see Fig. 2 ▸) is divided into three sections. In the first section, necessary modules for analysis and plotting are imported. A complete list of available modules can be found in the API documentation (see *Usage notes* [Sec sec4]). The second section defines the data source, using the 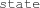 variable. In the third section, inside the function 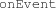, user-specific steps of analysis and plotting are performed, triggered by individual events. Global parameters can be defined outside the 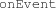 function, *e.g.* using the 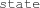 variable, which is used to keep track of things that do not change between consecutive 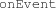 invocations.

### 2.2. Translation layer   

*Hummingbird*’s architecture (see Fig. 1 ▸) is designed such that all code parts downstream of the translation layer are intended to be facility agnostic. Native events are translated into *Hummingbird* events, represented by the event variable 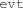. In its current implementation *Hummingbird* provides translation services for data produced at the LCLS using Python-based *psana* (Damiani *et al.*, 2016 ▸). This tool supports reading from the native XTC files at LCLS, as well as from live shared memory streams provided by the LCLS data acquisition infrastructure. The shared memory stream gives access to live LCLS data through a buffer which gets filled by the data acquisition system (DAQ) during data collection and is available to applications until it is overwritten by a subsequent event. The shared memory streams are only available from dedicated online monitoring nodes. In the future we plan to add more translation modules for other facilities like SACLA, and the upcoming European XFEL, which uses *Karabo* (Heisen *et al.*, 2013 ▸).

### 2.3. Event variable (evt)   

The 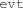 variable provides access to all available data entries, *e.g.* pixel detector images, pulse energies, motor positions. 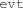 is a nested dictionary with two levels, the first defining a data type and the second defining a data key. Individual data records, like 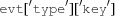 have 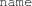 and 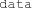 attributes, the former being an identifier 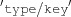 and the latter giving access to the data.

### 2.4. Analysis/plotting modules   

On the basis of their experimental needs, a user can customize individual data processing steps using analysis modules and define graphs to be presented in the GUI using plotting modules. An analysis module takes the 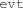 along with a data type and a data key as positional arguments. Some modules expect a data record as second argument. The output is attached to 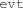 as a new data record with the data type 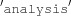 and a new data key based on the combination of the input data type/key and an additional string labelling the analysis performed. Additional input parameters can be passed to the module as a keyword argument.

A plotting module takes at least one data record as a positional argument and additional parameters as keyword arguments. The purpose of the plotting module is to send data together with plotting instructions to the interface. These plotting instructions include the kind of data to be plotted (*e.g.* image, histogram, scalar, vector,…), labels, limits and colormaps. Furthermore, it is possible to toggle a logarithmic mode, include horizontal/vertical markers and provide additional messages which are printed in the footer of the plotting window. Most of those plotting parameters can also be modified from the interface using the settings dialog inside the plotting window.

The current release of *Hummingbird* includes a collection of analysis modules for tasks such as detector correction, hit finding (Barty *et al.*, 2014 ▸), size and multiple-hit filtering (Hantke *et al.*, 2014 ▸), and scanning transmission X-ray microscopy analysis (for scanning experiments). The list of available plotting modules includes simple image and line plots as well as one-dimensional histograms and more advanced correlation plots (scatter plots, two-dimensional histograms).

The analysis modules are built upon *Numpy* (Jones *et al.*, 2001 ▸) and *Scipy* (van der Walt *et al.*, 2011 ▸).

### 2.5. Graphical user interface   

**Figure 3:**
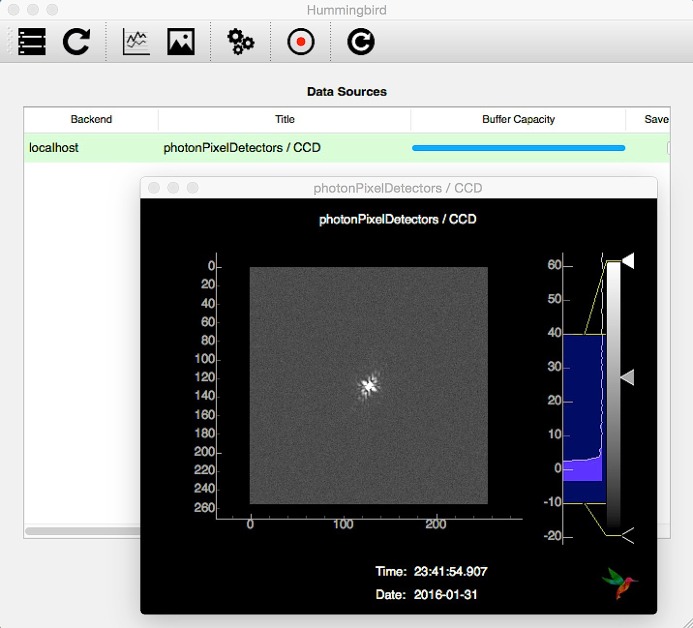
The GUI of the *Hummingbird* client. Once connected to the backend it shows a table of available data sources (in the background). On opening a line/image window (in the front) it is possible to subscribe to the data source and visualize data in real time as they are produced by the backend workers.

After connecting to the backend, the GUI provides a table of available data sources for plotting as shown in Fig. 3 ▸. This list is auto-populated on the basis of what is configured in the backend configuration file. Hence, no user modification of frontend source code is generally needed. Depending on the type of data source, data can be visualized in a line or image plot window.

After opening an empty line/image plot window it is possible to subscribe to available sources. At that point, the interface starts to subscribe to data from the backend workers, with plots being updated every other second. By closing the line/image window, the interface unsubscribes from the data source, minimizing network load. Multiple frontend processes on different machines can subscribe to the same backend without interfering with each other, allowing for specialized viewports.

For all data sources with active subscriptions, the interface keeps a buffer which can be dynamically resized at any time. Using this buffer, it is possible to show trends as well as go back in history in case an interesting event passed by too quickly. A more complete list of features is given in the GUI documentation (see *Usage notes* [Sec sec4]).

The GUI is written in Python using *Qt* (The Qt Company, 2016 ▸) through *PyQt* (Riverbank Computing, 2016 ▸) and *PyQtGraph* (Campagnola, 2016 ▸).

## 3. Practical example   

We demonstrate the usage and capability of *Hummingbird* using data from an FXI experiment on mimivirus collected at the LCLS (Ekeberg *et al.*, 2016 ▸). The dataset is deposited in the CXIDB (http://www.cxidb.org/; entry 30). A list of used raw XTC and index files is provided in Table 1 ▸.

**Figure 4:**
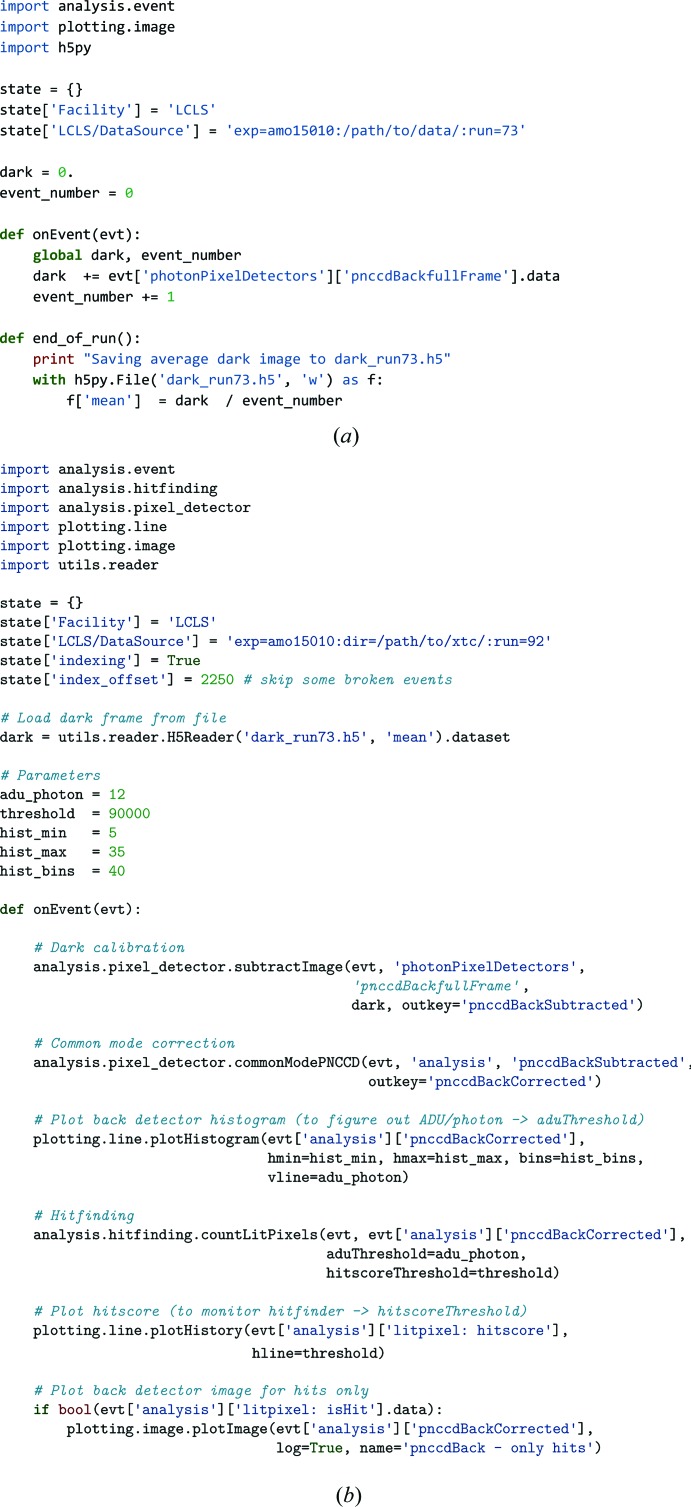
Example of *Hummingbird* configuration files reading data from raw XTC and index files. The configuration in (*a*) reads dark frames and saves the average to a file. The configuration in (*b*) reads diffraction frames, applies hit-finding analysis and sends detector images of hits along with additional information to the GUI client.

With all files listed in Table 1 ▸ in the same folder and a working *psana* setup, it is possible to run *Hummingbird* with the configuration described in Fig. 4 ▸. This example starts by calculating the average dark image, which then is subtracted from the raw pedestal values of diffraction frames. Running *Hummingbird* on the ‘dark’ configuration file (Fig. 4 ▸ *a*) in a single process will produce an HDF5 file with an averaged dark image.

When *Hummingbird* is run on the ‘diffraction’ configuration file (Fig. 4 ▸ *b*) in a single process, or with multiple processes, each worker grabs a raw frame from the back detector, subtracts the average dark image, corrects for the common mode within the pnCCD detector rows (Hantke *et al.*, 2014 ▸) and finally counts the number of lit pixels, *i.e.* pixels containing photons. We call this metric hitscore and use it to determine whether the current diffraction event is a hit or not. The backend sends three plots to the interface; the histogram of the current detector event, the current hitscore which is displayed as a history plot and the full detector image for hits.

**Figure 5:**
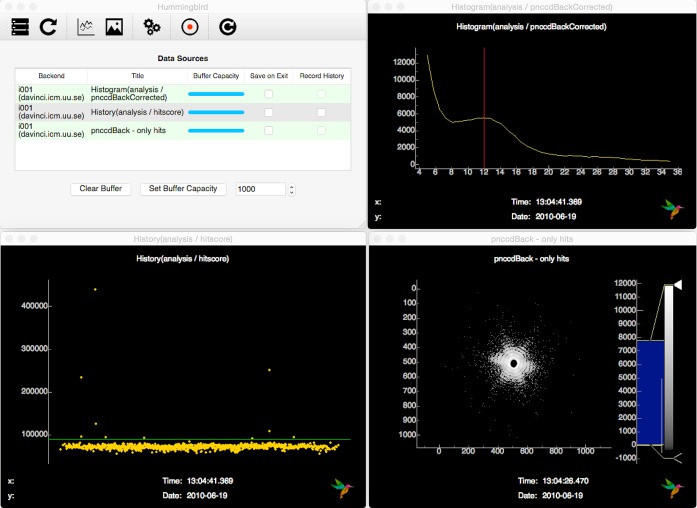
Snapshot of the *Hummingbird* interface showing available data sources and plots for the ‘diffraction’ configuration example. It monitors the hitscore (bottom left), detector histogram (top right) and latest hit image (bottom right). The current parameters of the hit finder (counting lit pixels) are shown as a vertical red line in the top right panel and as a horizontal green line in the bottom left panel.

After connecting the interface to the main worker of the backend, and subscribing to the three available data sources, updates on the histogram, hitscore and hit images are displayed in the interface as shown in Fig. 5 ▸. Watching the progress of histogram and hitscore history helps the user to optimize the lit pixel hit finder, namely the threshold for defining a lit pixel (shown as a vertical red line in the top right panel) and the hitscore threshold defining a hit (shown as a horizontal green line in the bottom left panel). When running from a dynamic data source (*e.g*. a shared memory stream), it is possible to change parameters in the backend configuration file and simply reload the configuration using the ‘Reload’ button in the interface without restarting the backend.

The performance of *Hummingbird* running the ‘diffraction’ configuration example when reading from XTC files is given in Table 2 ▸, showing processing rates above 100 Hz using up to 90 CPU workers distributed across five computing nodes. Each node has two Intel Xeon E5-2620 CPUs with six cores each. With low worker counts, the processing speed scales linearly. For higher counts, the disk I/O subsystem of our cluster becomes the limiting factor. When running the same example live (*i.e.* from shared memory streams at LCLS), the intent would be to choose a number of nodes (with the number of workers per node roughly corresponding to the number of cores) that would guarantee real-time processing of all data. During numerous experiments at the LCLS using similar configurations, *Hummingbird* has been reading from multiplexed shared memory streams approaching the real-time rate of 120 Hz.

## 4. Usage notes   

*Hummingbird* is an open-source project, available under the Simplified BSD license. The current release can be downloaded from http://lmb.icm.uu.se/hummingbird. The project is also available on Github (https://github.com/FXIhub/hummingbird). Detailed installation instructions and examples are provided on a documentation page (http://lmb.icm.uu.se/hummingbird/docs).

## 5. Future work   

In future releases of *Hummingbird* we are planning to add more event translators for other XFEL data sources, in particular for SACLA and the European XFEL. Besides its main focus on real-time monitoring, we are improving the capabilities of running *Hummingbird* as a tool for offline analysis of X-ray diffraction data. We continue to implement new data analysis and plotting modules. Furthermore, we are constantly adding new features to the GUI. This is an open-source project, so we encourage users to contribute and extend *Hummingbird* by adding new modules.

## 6. Conclusion   

We have introduced *Hummingbird*, a versatile data analysis and monitoring tool which is able to cope with the current frame rates at which data are produced in FXI experiments performed at free-electron lasers. With *Hummingbird*, users are able to monitor and thus adjust crucial experimental parameters in real time. With its simple and modular Python implementation, users can easily build their own tailored analysis pipeline for their experiments and run it in real time over multiple cores and nodes.

## Figures and Tables

**Table 1:** List of raw XTC and index files downloaded from the CXIDB (entry 30): files containing dark frames (left column) and diffraction frames (right column) are used for demonstration of *Hummingbird*

Run 73 (dark)	Run 92 (diffraction)
e41-r0073-s00-c00.xtc	e41-r0092-s01-c00.xtc
e41-r0073-s02-c00.xtc	e41-r0092-s00-c00.xtc
e41-r0073-s01-c00.xtc	e41-r0092-s02-c00.xtc
index/e41-r0073-s00-c00.xtc.idx	index/e41-r0092-s01-c00.xtc.idx
index/e41-r0073-s02-c00.xtc.idx	index/e41-r0092-s00-c00.xtc.idx
index/e41-r0073-s01-c00.xtc.idx	index/e41-r0092-s02-c00.xtc.idx

**Table 2:** Processing rates of *Hummingbird* running with MPI on the configuration file shown in Fig. 4 ▸ using different numbers of workers

No. of workers	Processing rate (Hz)		No. of workers	Processing rate (Hz)
1	3.93		50	141.99
10	37.69		60	155.36
20	57.50		70	178.53
30	89.20		80	189.74
40	110.29		90	198.26
